# Supplementary figures and images for: Screen Printing Carbon Nanotubes Textiles Antennas for Smart Wearables
Source: Sensors (Basel). 2021 Jul 20;21(14):4934. doi: 10.3390/s21144934 (PMC8309715; doi:10.3390/s21144934)

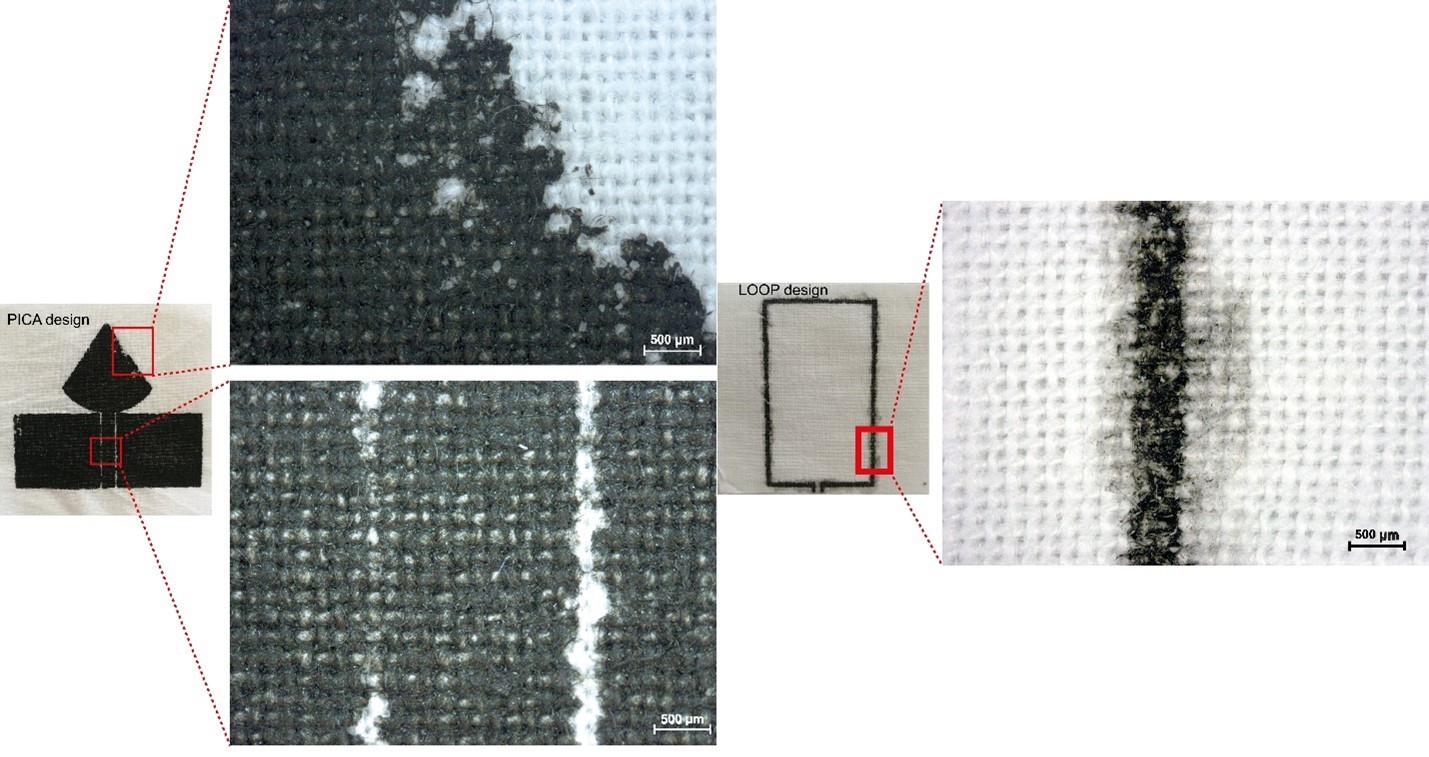

Supplement: Supplementary file 1 [file sensors-21-04934-s001.zip › Supplementary Images/Figure S1.jpg]

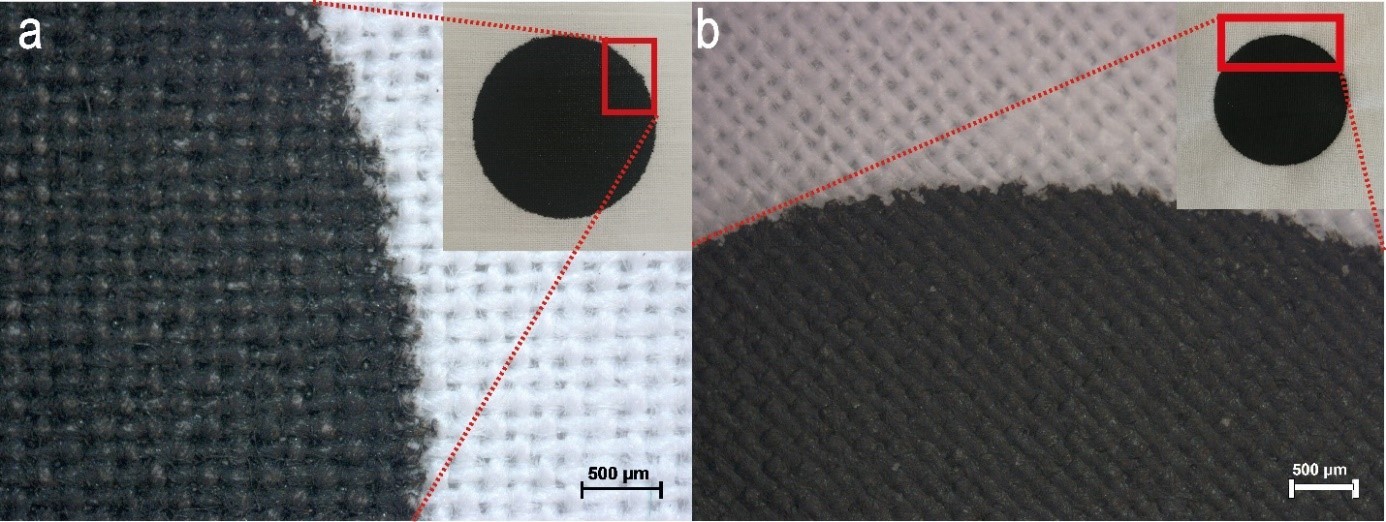

Supplement: Supplementary file 1 [file sensors-21-04934-s001.zip › Supplementary Images/Figure S2.jpg]
